# Metal-Sulfate Induced Generation of ROS in Human Brain Cells: Detection Using an Isomeric Mixture of 5- and 6-Carboxy-2′,7′-Dichlorofluorescein Diacetate (Carboxy-DCFDA) as a Cell Permeant Tracer

**DOI:** 10.3390/ijms13089615

**Published:** 2012-08-02

**Authors:** Aileen I. Pogue, Brandon M. Jones, Surjyadipta Bhattacharjee, Maire E. Percy, Yuhai Zhao, Walter J. Lukiw

**Affiliations:** 1 Alchem Biotek Corporation, Toronto, ON M5T 1L8, Canada; E-Mail: wlukiw@yahoo.com; 2 LSU Neuroscience Center and Department of Ophthalmology, Louisiana State University Health Sciences Center, New Orleans, LA 70112, USA; E-Mails: bjone9@lsuhsc.edu (B.M.J.); sbhat3@lsuhsc.edu (S.B.); 3 Surrey Place Centre, University of Toronto, Toronto, ON M5S 1A8, Canada; E-Mail: maire.percy@utoronto.ca; 4 University of Texas Health Science Center, Houston, TX 77030, USA; E-Mail: yzhao4@lsuhsc.edu

**Keywords:** 5-carboxy-2′,7′-dichlorofluorescein diacetate, 6-carboxy-2′,7′-dichlorofluorescein diacetate (5- and 6-carboxy-DCFDA; carboxy-DCFDA), aluminum, Alzheimer’s disease, amyotrophic lateral sclerosis, carboxy-DCFDA, epigenetic human neural cells, inflammation, metal sulfates, synergistic effects, Parkinson’s disease, prion disease

## Abstract

Evolution of reactive oxygen species (ROS), generated during the patho-physiological stress of nervous tissue, has been implicated in the etiology of several progressive human neurological disorders including Alzheimer’s disease (AD) and amylotrophic lateral sclerosis (ALS). In this brief communication we used mixed isomers of 5-(and-6)-carboxy-2′,7′-dichlorofluorescein diacetate (carboxy-DCFDA; C_25_H_14_C_l2_O_9_; MW 529.3), a novel fluorescent indicator, to assess ROS generation within human neuronal-glial (HNG) cells in primary co-culture. We introduced pathological stress using the sulfates of 12 environmentally-, industrially- and agriculturally-relevant divalent and trivalent metals including Al, Cd, Cu, Fe, Hg, Ga, Mg, Mn, Ni, Pb, Sn and Zn. In this experimental test system, of all the metal sulfates analyzed, aluminum sulfate showed by far the greatest ability to induce intracellular ROS. These studies indicate the utility of using isomeric mixtures of carboxy-H_2_DCFDA diacetates as novel and highly sensitive, long-lasting, cell-permeant, fluorescein-based tracers for quantifying ROS generation in intact, metabolizing human brain cells, and in analyzing the potential epigenetic contribution of different metal sulfates to ROS-generation and ROS-mediated neurological dysfunction.

## 1. Introduction

**Figure 1:**
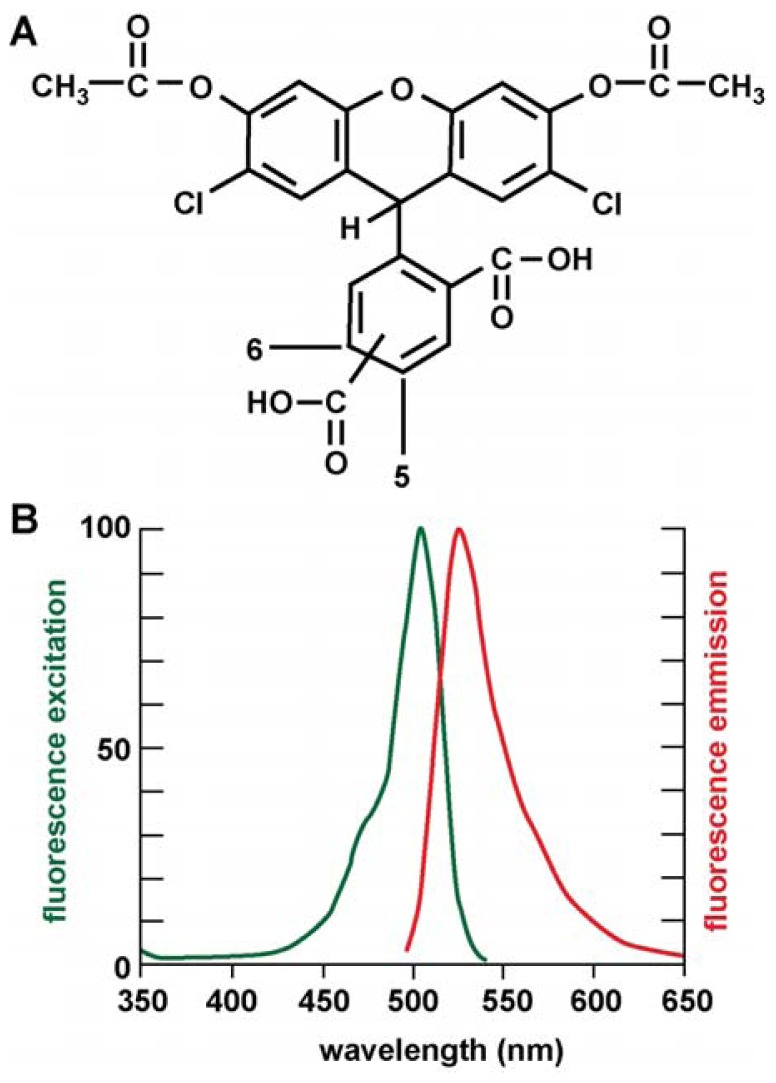
(**A**) Molecular structure of the mixed isomer, cell permeant 5-(and-6)-carboxy-2′,7′-dichlorofluorescein diacetate [carboxy-DCFDA; C_25_H_14_C_l2_O_9_; MW 529.3; Molecular Probes C-369; CAS name = 3′,6′-bis(acetyloxy)-2′,7′-dichloro-3-oxo-spiro-[iso-benzofuran-1(3*H*),9′-(9*H*)xanthene-ar-carboxylic acid; CAS number 127770-45-0]; the twin CH_3_COO-R-groups facilitate cellular entry; intracellular esterases cleave these to “trap” the molecule within the cell; (**B**) peak excitation (λE_x_ 502 nm; shown in green) and peak emission (λE_m_ 530 nm; shown in red) for 5-(and-6)-carboxy-2′,7′-dichlorofluorescein diacetate after removal of acetyl groups by cellular esterases; the dicarboxyl groups at positions 5 and 6 appear to stabilize the carboxy-DCFDA flurophor to prolong intracellular fluorescence yield.

The evolution of reactive oxygen species (ROS) is a persistent and ongoing metabolic process during the course of normal human aging. Excessive ROS generation that overwhelms cellular anti-oxidant defenses, and ensuing free-radical damage to cellular lipids, proteins, and nucleic acids, lies at the core of the widely accepted free radical theory of aging [1–4]. This theory proposes that aging is accompanied by increased ambient levels of mitochondrial- and microglial-mediated, inflammation-generated ROS resulting in oxidized and super-oxidized molecules, biological signaling defects and deleterious effects on the maintenance of normal cellular homeostasis [1–8]. Indeed the pathological overproduction of ROS, in excess of that which the endogenous antioxidant systems of cells can handle, has been implicated in the development of numerous sporadic and progressive human neurological disorders including Alzheimer’s disease (AD), Parkinson’s disease (PD), amylotrophic lateral sclerosis (ALS) and prion disease [8–13]. Brain cells may be particularly sensitive to free radical and oxidative stress because of their high intrinsic metabolic rates, and the high complexity of genetic activity within their nuclei [12–16]. One of the strongest exogenous sources of free-radical stress are trace metal sulfates from environmental or industrial exposure [8,14]. As an initial step to quantify and understand ROS-inducibility by relatively common metal sulfates, in these experiments we studied the ROS-inducing capabilities of Al, Cd, Cu, Fe, Hg, Ga, Mg, Mn, Ni, Pb, Sn and Zn (as sulfates) at 50 nanomolar concentrations in human neuronal-glial (HNG) cell co-cultures using a novel, mixed isomer, fluorescent indicator 5-(and-6)-carboxy-2′,7′-dichlorofluorescein diacetate (carboxy-DCFDA; CDCFDA; C_25_H_14_C_l2_O_9_). This cell membrane-permeable, fluorescein-based indicator has significant advantages over previous fluorescein diacetate-based tracer systems in that it rapidly and efficiently diffuses into cells as a colorless, non-fluorescent probe until the two acetate groups are cleaved by intracellular esterases to yield the fluorescent fluorophore, 5-(and-6)-carboxy-2′,7′-dichlorofluorescein [17]. Once internalized, the 5-(and-6)-carboxy-2′,7′-dichlorofluorescein isomers cannot efficiently exit the cell, resulting in significantly longer signal reporting times as detected by fluorescent microscopy and electronic signal capture (λE_x_ 502 nm; λ_m_ 530 nm; Figure 1) [14,17]. Moreover, carboxy-DCFDA has exceedingly low sensitivity of intrinsic fluorescence yield at intracellular pH, and the 5′-and 6′-carboxy-ligands significantly further stabilize the internalized fluorescent signal enabling longer data-collection times. Using this novel and highly sensitive analytical tracer in this study we have quantified the ROS-producing capability of 12 environmentally- and industrially-relevant metal sulfates in human neuronal-glial (HNG) cells in primary culture. HNG cells represent a previously verified, highly sensitive and physiologically relevant *in vitro* stress-test analytical system for determining the potential of specific metal sulfates to contribute, via ROS generation, to human neurological diseases with an oxidative stress component [6–15].

## 2. Results

**Figure 2:**
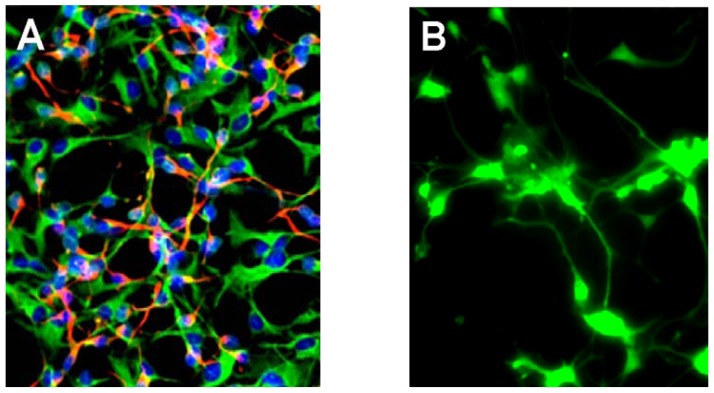
(**A**) Human neuronal-glial (HNG) cells after 2.5 weeks in primary co-culture; the cell density is approximately 35% neurons and 65% astroglia at 60% confluency; human primary neuronal and glial “support” cell co-cultures are used as human neuronal cells do not culture well by themselves [19,22]; neuronal cells are stained with neuron-specific β-tubulin (red; λ_max_ = 690 nm), glial cells are stained with glial-specific glial fibrillary acidic protein (GFAP; green; λ_max_ = 525 nm), and nuclei are stained with Hoechst 33258 (blue; λ_max_ = 470 nm); photo magnification 20×; (**B**) co-incubation with 5-(and-6)-carboxy-2′,7′-dichloro-fluorescein diacetate (C_25_H_14_C_l2_O_9_; carboxy-DCFDA) indicates appreciable ROS generation throughout the entire neuronal-glial cell soma and neurite extensions in all cell types; 100% of the cells are stained and exhibit varying degrees of ROS generation depending on anatomical location; treatment shown after 3 h with 50 nM Al_2_(SO_4_)_3_ displays significant ROS signal yield with a green fluorescence emission λ_max_ 530 nm (Figure 1); photo magnification 30×.

The molecular structure of carboxy-DCFDA is shown in Figure 1(A) and the fluorescence excitation and emission spectrum for carboxy-DCFDA is shown in Figure 1(B). A typical 2.5 week old culture of HNG cells is shown in Figure 2(A), and a typical carboxy-DCFDA-based ROS assay is shown in Figure 2(B). Fluorescent signals from stressed HNG cells were quantified using digital electronic imaging photography under ultraviolet (UV) light (E_x_ 502 nm; E_m_ 530 nm) employing an Axioskop/Zeiss MC63 photo control unit and a Nikon Optiphot-2 microscope equipped with an additional differential-Interference Contrast/Nikon UFX–DX photo control unit.

The ROS signal intensity for the 12 metal sulfates, plus the additional control Na_2_(SO_4_), tested in these experiments are shown in Table 1. Depending on the extent of ROS generated a semi-quantitative scale of 1 through 10 was electronically derived from the total ROS raw signals obtained from the same density of control and metal-sulfate treated HNG cells as previously described [8,14,18–21]. Briefly, HNG cells treated with MgSO_4_, showed very minimal, if any, generation of ROS above control [Na_2_(SO_4_)] values. Using the novel carboxy-DCFDA-based ROS assay the order of effectiveness of metal sulfates to generate ROS was Al >> Fe >> Mn > Zn > Ni > Pb > Ga > Cu > Cd > Sn > Hg > Mg. These results are in agreement with a previously published report that tested the ROS-generating efficacy of 6 metal sulfates using the non-carboxylated fluorescent indicator 2′,7′-dichlorofluorescein diacetate (H_2_DCFDA) [8,10,11]. The persistence of carboxy-DCFDA fluorescence yield in HNG cells was found to be at least 10-fold longer than H_2_DCFDA using the same human brain cell types and the same analytical conditions [7,8,14].

## 3. Discussion

The major experimental focus of these studies was to characterize the relative ROS-generating capability of physiologically-relevant, environmental and industrial metal sulfates using HNG cells in primary co-culture, using the novel dual ROS sensors 5-carboxy-DCFDA and 6-carboxy-DCFDA in an equimolar mixture. HNG cells have previously provided a proven primary human brain cell analytical assay that is both representative of the two major human neocortical brain cell types, and are very highly sensitive (more so than mouse or rat brain cells) to exogenous or epigenetic, physiologically-relevant ionic or molecular stressors [8,14–16,18–32]. Indeed, it is well documented that HNG cells in primary culture are exquisitely sensitive to externally applied stressors in the low nanomolar range, and that excessive ROS generation in brain cells and CNS tissues rapidly promotes cellular oxidative stress that progressively renders normally functioning DNA, lipids, proteins and RNA incapable of performing their homeostatic metabolic and cell-signaling functions. These ideas have been interpreted to support of the free-radical theory of aging [1,4,5,10,11,14–16,27–32]. Aging is the greatest known risk factor for the onset of neurodegenerative diseases such as sporadic Alzheimer’s disease (AD), amyotrophic lateral sclerosis (ALS) and related, progressive, incurable neurological disorders including Parkinson’s disease (PD), prion disease and others [4–14,26]. In human nervous tissue, both mitochondrial dysfunction and microglial-mediated inflammatory processes increase with age, and increased production of ROS and oxidative stress is highly damaging to both neurons and glia in these progressive, age-related human neurodegenerative conditions [18–20,26,27]. Moreover the experimental use of anti-oxidants and free radical trapping agents have shown significant benefit in quenching neurotoxic metal effects by reducing oxidative stress and ROS generation in both these *in vitro* test systems and also in human clinical trials [15,17,20,28].

These findings further underscore the idea that environmentally- and industrially-relevant trace metals, at physiologically realistic, low nanomolar concentrations are highly effective in inducing ROS. Highly complex mixtures of neurotoxic metals in combination with cytokines and pathological peptides, as might be expected to occur in vivo, appear to induce synergistic effects in promoting stress and neurodegeneration [8,14,20,21]. Further, ROS is a potent inducer of the pro-inflammatory transcription factor NF-κB, and NF-κB-regulated biological targets including, for example, the inflammatory cytokine tissue necrosis factor alpha (TNFα) as well as various pro-inflammatory micro RNAs miRNAs [21,33–39]. As an established ROS-responsive transcription regulator NF-κB is up-regulated in virtually every human neurological disorder so far examined, so that besides anti-oxidant approaches, selective NF-kB inhibitors and specific chelators with innovative chelation or competition strategies may be useful to neutralize the initial effects of neurotoxic metal-sulfates in human brain cells [20,31–42].

The metal sulfates studied in this report all have considerable industrial, manufacturing and agricultural applications, and all are toxic to various degrees, in various biological systems, depending on concentration and bioavailability [8,14,43–45]. For example, aluminum-, copper- and zinc-sulfates are widely used as herbicides, fungicides, pesticides and molluscicides, suggesting a toxicity even to the most widespread and resilient organisms in the biosphere [40,43–45]. Besides being toxic to the human reproductive system, mucous membranes, skin, eyes, and urinary system, aluminum sulfate is intensely genotoxic [46–48]. Interestingly, aluminum sulfate is a common, highly soluble, additive, as alum [Al_2_(SO_4_)_3_ or KAl(SO_4_)_3_], used worldwide to treat waste water and to clarify turbid drinking water to give water a clear “finished” appearance [49,50]. Although aluminum sulfate, as other aluminum salts, have relatively limited bioavailability, there are consistent reports from multiple, independent sources that the aluminum or alum content of drinking water impacts AD incidence [49,50]. Similarly excessive environmental accumulation of Mn in the human basal ganglia results in a neurological syndrome with cognitive, psychiatric, and movement abnormalities characteristic of Parkinsonism with clinical features similar to those of Parkinson’s disease (PD) [51].

Lastly, it should be mentioned that these studies have several important limitations. The age of HNG cells has some bearing on the extent of oxidative stress response and hence ROS generation; older, more differentiated cells are generally less responsive to metal-sulfate induced ROS production than younger cell cultures [8,14,15]. While a 50 nM dosage of metal sulfates has been previously shown be effective in ROS induction in a wide variety of brain cell types, other concentrations or combinations of metal sulfates may be additive or synergistic in ROS induction efficiency [8,14]. Indeed, this current study represents a significant improvement in ROS detection sensitivity in an extremely metabolically and genetically active cell type, carboxy-DCFDA may not detect all forms of ROS including rare and exotic ROS or reactive nitrogen species (RNS) [3,48,52]. Complex combinations of metal sulfates with other pathological molecules such as cytokines and amyloid peptides, as might be expected under real life physiological conditions, have not been adequately explored, and their ROS-inducing capabilities require further study.

## 4. Experimental Section

### 4.1. Reagents and Antibodies

All ROS-generating metals were used as ultrapure sulfates, in part, as previously described [8,14]. Briefly, Biochemika MicroSelect^©^ ultrapure reagents for molecular biology, including Al_2_(SO_4_)_3_ (11044), Cd(II)SO_4_ (481882), Cu(II)SO_4_ (35185), Fe(II)SO_4_ (44970), Ga_2_(SO_4_)_3_ (463892), Hg(II)SO_4_ (83372), Mg(II)SO_4_ (63133), Mn(II)SO_4_ (31425), Ni(II)SO_4_ (656895), Pb(II)SO_4_ (254258), Sn(II)SO_4_ (96555) and Zn(II)SO_4_ (35392; Sigma-Aldrich or Fluka Chemical, Milwaukee, WI, USA), were freshly prepared as 0.1 M stock solutions, and were instilled into serum-containing HNG cell maintenance medium (HNGMM, pH 7.5; see section below for details) by gentle inversion, followed by filter sterilization using 0.2-μM disposable spin filters (Millipore Corporation, Billerica, MA, USA) [8,14,15]. All metal sulfate solutions were used at 50 nM concentrations in HNGMM (Table 1). HNG cells, HNGMM and bullet packs containing human epidermal and fibroblast growth factor (E/FGF), gentamicin/amphotericin (G/A1000), neural survival factor-1 (NSF-1) and FBS were obtained from Lonza (Walkersville, MD, USA). All other reagents were of the highest ultrapure grades commercially available and were used without further purification [8,14–16].

### 4.2. Ultrapure Water and Minimization of Extraneous Contamination

Throughout the experimental work ultrapure water (18 megohm, Milli-Q, Millipore or Puriss 95305, Fluka) was employed in all cell culture and biochemical procedures to stringently exclude trace metal extraneous contamination; as analyzed by electrothermal atomic absorption spectroscopy, aluminum, copper, magnesium, manganese, mercury, iron, tin and zinc sulfate content were ≤10 ppb. Coded isolation reagent and media samples were analyzed for potential trace metal contamination using a Perkin Elmer 5000PC Zeeman-type electrothermal atomic absorbance (EAA) spectrophotometer equipped with an automated sampler and IBM/AT-supported analysis package for trace metal analysis [8,14–16,18–21]. Wherever possible, ultrapure HNO_3_ washed polysulfone plasticware was used according to the URI-GSO protocols to stringently eliminate exogenous trace metal contamination [8,14].

### 4.3. Human Neuronal-Glial (HNG) Cells in Primary Culture

HNG cell lines, derived from cryopreserved normal human neural progenitor cells (PT-2599; Lonza-Clonetics Cell Systems, Walkersville, MD, USA) were cultured in 6-well (3.5 cm diameter) plates (Costar 3506, Corning Life Sciences, Acton, MA, USA) at 5% CO_2_, 20% O_2_ and 37 °C in HNGMM supplemented with 2.5% serum containing hFGF (human fibroblast growth factor), NSF-1 (neuronal survival factor 1), hEGF (human epidermal growth factor) and GA-1000 (gentamicin-amphotericin B G/A 1000) as previously described [18,19,21,22–25]. HNGMM was completely changed every 3 days of culture. At 2.5 weeks of growth there were approximately 35% neurons and 65% astroglia (Figure 2) at 55% cell confluency. HNG cells were screened to be free from transmissible pathogens (HIV, HSV-1, *etc.*) at source, tested negative for microglial, endothelial or fibroblast cell markers, and tested positive only for the nuclear-, neuronal- and glial-specific markers Hoechst 33258, bTUBIII and GFAP, respectively [18–25].

### 4.4. ROS Assay Using the Novel CDCFDA [5-(and-6)-darboxy-2′,7′-Dichlorofluorescein Diacetate] “Mixed Isomers”

The abundance of reactive oxygen species (ROS) was assayed in metal-sulfate-treated, Na_2_SO_4_-treated (control) or un-treated 2.5 weeks old HNG cells (Figure 2) using an equimolar mixture of 5-carboxy-2′,7′-dichlorofluorescein diacetate (5-CDCFDA) and 6-carboxy-2′,7′-dichlorofluorescein diacetate (6-CDCFDA; soluble in dimethyl sulfoxide; collectively known as carboxy-DCFDA; Figure 1) at a 10 μM ambient concentration in cell culture medium using protocols provided by the manufacturer (Molecular Probes-Invitrogen, Carlsbad, CA, USA) and as previously described for 2′,7′-dichlorofluorescein diacetate (H_2_DCFDA) [8,14]. After cell entry and hydrolysis by non-specific intracellular esterases, 5-CDCFDA and 6-CDCFDA react with singlet oxygen, hydroxyl radicals or superoxide-generating green fluorescent signals (collectively termed ROS) that are quantified using electronic imaging photography (E_x_ 502 nm; E_m_ 530 nm; Figure 1) using a Zeiss Axioskop/Zeiss MC63 photo control unit coupled to a Nikon Optiphot-2 microscope equipped with an additional differential Interference Contrast/Nikon UFX-DX photo control unit [8,14].

### 4.5. Statistical Analysis

All electronically imaged data was imported into an Intel Pentium 6, 6.4 GHz dual processor computer and graded on a scale from “0 to 10” ROS yield, *i.e.*, zero fluorescence (*i.e.*, after treatment with Na_2_SO_4_) compared to maximum fluorescence (*i.e.*, after treatment with Al_2_S(SO_4_)_3_ (Table 1) [8,14]. Statistical procedures and analysis were carried out using the programs and procedures in the SAS language (Statistical Analysis System, SAS Institute: Cary, NC, USA, Year). All *p* values were derived from protected *t*-tests or least square means from a two-way factorial analysis of variance (*p*, ANOVA); only *p*-values of less than 0.05 were considered to be statistically significant.

## 5. Conclusions

The dual ROS sensors, 5-carboxy-DCFDA and 6-carboxy-DCFDA, described in these experiments should be useful to compare the effects of other physiologically relevant stressors, chelators, and other inhibitory molecules, to further our understanding of metal sulfate-mediated, ROS-initiated pathogenetic signaling that ultimately contributes to progressive neurodegenerative events in brain cells.

## Figures and Tables

**Table 1:** Effects of different metal sulfates as physiological stressors, at 50 nM concentrations, on reactive oxygen species (ROS) generation in human neuronal-glial (HNG) primary cell cultures [8,14]. Note: ROS intensity [raw signal at an emission λ_max_ of 530 nm (Em 530 nm)] refers to mean relative raw digitized electronic signal yield at 530 nm obtained from the Zeiss Axioskop/Zeiss MC63 photo control unit; accordingly metal sulfates were stratified by their intrinsic capability to generate ROS by methods previously described [8,14]; each metal sulfate effect on ROS generation was assayed three times; a scale of 1–10 was derived on these 13 evaluations as well as from previous reports [8,14].

Metal sulfate	ROS intensity (raw signal at Em 530 nm)	Relative induction of ROS
Na	1	0
Mg	1.2	0
Hg	18	1.5
Sn	26	2
Cd	36	3
Cu	36	3
Ga	36	3
Pb	42	3.5
Ni	42	3.5
Zn	48	4
Mn	53	4.5
Fe	73	6
Al	121	10
